# Anti-Tumor Role of CAMK2B in Remodeling the Stromal Microenvironment and Inhibiting Proliferation in Papillary Renal Cell Carcinoma

**DOI:** 10.3389/fonc.2022.740051

**Published:** 2022-01-21

**Authors:** Qingan Jia, Xia Liao, Yaoyao Zhang, Binghui Xu, Yuna Song, Ganlan Bian, Xiaoliang Fu

**Affiliations:** ^1^ Xi’an Key Laboratory of Stem Cell and Regenerative Medicine, Institute of Medical Research, Northwestern Polytechnical University, Xi’an, China; ^2^ Department of Nutrition, First Affiliated Hospital of Xi’an Jiaotong University, Xi’an, China; ^3^ Department of Urology, The Second Affiliated Hospital of Air Force Medical University, Xi’an, China

**Keywords:** kidney renal papillary cell carcinoma (KIRP), tumor microenvironment, CAMK2B, lncRNA GUSBP11, miR-432-5p

## Abstract

The tumor microenvironment (TME) is variable across tumor types and has diverse effects on malignant progression, based on the type and number of infiltrating stromal cells. In particular, TME effector genes and their competitive endogenous RNA (ceRNA) networks play a critical role in regulating malignant tumor progression. However, the core effector molecules involved in TME modulation of kidney renal papillary cell carcinoma (KIRP) are poorly understood. To address this question, a cohort containing 233 KIRP patients was derived from The Cancer Genome Atlas (TCGA) database, and the data were processed using the ESTIMATE algorithm. We further evaluated the relationship between immune scores (ISs) and stromal scores (SSs) and disease progression and found that high SSs were associated with a poor prognosis in KIRP. Differentially expressed genes (DEGs) were therefore screened based on SS scores, resulting in 2509 DEGs, including 1668 mRNAs, 783 long noncoding (lnc)RNAs, and 58 micro (mi)RNAs. DEGs were then filtered using the random variance and subjected to hierarchical clustering using EPCLUST. Weighted gene co-expression network analysis (WGCNA) was used to assess the prognostic capacity of these DEGs and identify target ceRNA networks, and lncRNA GUSBP11/miR-432-5p/CAMK2B in the turquoise module was selected as a promising ceRNA network. From this analysis CAMK2B was selected as the core gene predicted to be involved in stromal TMA regulation. We therefore explored the expression and function of CAMK2B *in vitro* and *in vivo* and provide evidence that this protein promotes stromal TME remodulation and inhibits proliferation in KIRP. Lastly, we show that vascular endothelial growth factor (VEGF), transforming growth factor (TGF)β, and close homolog of L1 (CHL1) act as downstream effectors of CAMK2B in KIRP. Thus, in this study, we show that the TME determines prognosis of KIRP patients *via* the core effector molecule CAMK2B, which mediates both microenvironmental remodeling and tumor progression. Based on these findings, we propose that remodeling of the stromal microenvironment could represent an improved therapeutic approach relative to immunotherapy for KIRP.

## Introduction

According to estimates from the World Health Organization (WHO) and Chinese National Cancer Center, cancer is the leading cause of death and consequently, represents a major public health problem in a majority of countries, showing increasing incidence and mortality rates (1, 2). Kidney renal papillary cell carcinoma (KIRP) accounts for 10–20% of Renal Cell Carcinoma (RCC). However, efficacy data for drugs available in clinical practice remain largely unsatisfactory for KIRP, as patients with KIRP are often excluded from common RCC histological subtypes in clinical trials. Furthermore, the regulatory factors that mediate KIRP progression remain largely unidentified (3, 4). In recent years, the emergence of immune checkpoint inhibitors and combination treatment strategies has brought hope to patients with advanced RCC. However, additional insight into the driver and passenger alterations in KIRP-associated genes will be key to improving both management of this disease and patient prognosis.

The tumor microenvironment (TME) strongly influences clinical outcome of patients with malignant tumors, and the TME landscape varies considerably in different tumor types (5). Critically, characteristic features of the TME and the precise effect of key TME-associated genes in KIRP remain unclear. Emerging studies suggest that noncoding competing endogenous RNAs (ceRNAs), including microRNAs (miRNAs) (6) and long noncoding RNAs (lncRNAs) (7), play a critical role in the efficiency of messenger RNA (mRNA) translation in tumors (8). These noncoding RNAs modulate gene expression at both the transcriptional and post-transcriptional levels and act as master regulators for key mRNAs. However, the target genes and related effector proteins involved in TME-mediated modulation of KIRP are poorly understood.

In the present study, we aimed to explore the role of TME subtype in malignant progression of KIRP and to identify the core effectors involved in KIRP progression. Unexpectedly, we found that overall survival was associated with stromal scores (SSs), but not immune scores (ISs), derived using the ESTIMATE algorithm. We then identified and screened differentially expressed genes (DEGs) in patients with high *vs.* low SSs using weighted gene co-expression network analysis (WGCNA), leading to identification of the lncRNA GUSBP11/MiR-432-5p/CAMK2B network, based on the results of correlation strength and survival analysis. Lastly, we investigated the role of CAMK2B, a core network gene, and found that it inhibits proliferation and modulates the TME *via* downstream effector genes, including vascular endothelial growth factor (VEGF), transforming growth factor (TGF)β, and close homolog of L1 (CHL1), which mediate in stromal microenvironment regulation. Thus our findings suggest that TME remodeling may represent a possible therapeutic approach for KIRP.

## Materials and Methods

### Data Acquisition and Estimation of Stromal Scores and Immune Scores

The Cancer Genome Atlas (TCGA) database contains multi-omics datasets, including gene transcript, miRNA, and lncRNA expression data from numerous cancer types. We downloaded all KIRP datasets from TCGA (https://portal.gdc.cancer.gov/) using the TCGAbiolinks package in R software (version 3.5.1), including lncRNA, mRNA, and miRNA expression profiles from KIRP samples and the corresponding clinical follow-up data. Next, we used the ESTIMATE algorithm to output stromal scores (SSs) and immune scores (ISs) and compared the differentially expressed lncRNAs, mRNAs, and miRNAs in samples with high and low SSs and ISs (high >0 and low <0) (9).

### Weighted Gene Co-Expression Network Analysis

We utilized the weighted gene co-expression network analysis (WGCNA) package in R to analyze the co-expression network of selected lncRNAs and mRNAs (10). The gene expression profile matrix was first transformed into a similarity matrix based on the Pearson test between pairwise genes. Next, the similarity matrix was converted into an adjacency matrix, and scale-free gene co-expression networks were constructed by the WGCNA package. The topological overlap matrix (TOM) and dissimilarity TOM (dissTOM) were then obtained by TOM similarity and dissimilarity on the basis of the adjacency matrix. Lastly, after hierarchical clustering analysis based on dissTOM, modules were generated from the Dynamic Tree Cut method for Branch Cutting.

### Tissue Samples and Clinical Data Collection

Two independent cohorts of KIRP samples were acquired for this study from patients that underwent surgery at the Air Force Military Medical University Tangdu Hospital (Xi’an, China). From cohort A, 30 paired KIRP tissues and adjacent non-tumor tissues were gathered and used for reverse transcription (RT)-PCR and immunoblotting. From cohort B, 80 KIRP specimens were collected and used for constructing tissue microarrays and performing immunohistochemistry (IHC). This study was approved by the Ethics Committee on Human Research of Tangdu Hospital, and written informed consent was obtained from all patients.

### Vector Construction, Transfection, and Lentivirus Transduction

Human full-length CAMK2B cDNA (NM_001220.5) was obtained from VectorBuilder (Guangzhou, China). This fragment was cloned into the pCDH lentiviral expression vector (System Biosciences, Palo Alto, CA, USA) and inserted between the *Xba*I and *Eco*RI sites, using the In-Fusion HD Cloning Kit (Takara, Tokyo, Japan). Plasmid constructs expressing *CAMK2B* shRNA and scramble shRNA were generated using the lentiviral expression plasmid PLKO.1 and obtained from Vectorbuilder. Lentiviral shRNA target sequences were as follows: CCGGAAGCAGGAGATCATTAA (sh1), GACCAGATGTGATTTGTTAAA (sh2), and ATAGAGGATGAAGACGCTAAA (sh3). Stable cell lines were created by antibiotic selection with puromycin for one week, beginning 72 h after transduction.

### Quantitative RT-PCR Analysis

Total RNA was isolated from KIRP tissues and adjacent non-tumor tissues using TRIzol Reagent (Invitrogen, Thermo Fisher Scientific, Waltham, MA, USA). Isolated RNA was reverse transcribed using the PrimeScript RT Reagent Kit (TaKaRa, Shiga, Japan), and mRNA expression was determined by quantitative (q) qRT-PCR using SYBR Premix Ex Taq II (TaKaRa). Primers used for amplification of human genes were as follows: lncRNA GUSBP11 forward primer 5’-TCCCCTGTCCCGAAGGATTAC-3’ and reverse primer 5’ -TAAGGGACTAACGGCTTCGCT-3’; miR-432-5p forward primer 5’-ACTCAAACACTTCGGACATGG-3’ and reverse primer 5’-CAAAGAGCAACAGAGAGTAGCA-3’; *CAMK2B* forward primer 5’-CAGTGTACTTCAGTTGGTG-3’ and reverse primer 5’-TCAGGTTTTGCTCTTC TC-3’; *GAPDH* forward primer 5’-AGGTCGGAGTCAACGGATTTG-3’ and reverse primer 5’-TGTAAACCATGTAGTTGAGGTCA-3’.

### Immunohistochemistry and Immunoblotting

Tumor tissue was fixed, embedded in paraffin, and sliced into 5-μm thick sections. IHC staining and immunoblotting were performed using primary antibodies to CAMK2B (1:100 dilution, Proteintech, Chicago, IL, USA), according to standard protocols, as previously described (11). Relevant bioinformatics methods, Culture conditions for cell lines, and the various functional assays for CAMK2B *in vivo* (animal models with subcutaneous xenografts) and *in vitro* (migration, proliferation, and antibodies and cell lines information) are described in the **Supplementary Materials and Methods (Doc. S1)**.

### Statistical Analysis

Statistical analyses were performed using SPSS 15.0 for Windows (SPSS, Inc., Chicago, IL, USA). IHC and qRT-PCR experiments were performed at least three times, and statistical quantitative data were evaluated by Student’s *t*-tests, with *P* <0.05 considered statistically significant.

## Results

### High SSs Are Associated With Poor Prognosis in KIRP

**Figure 1:**
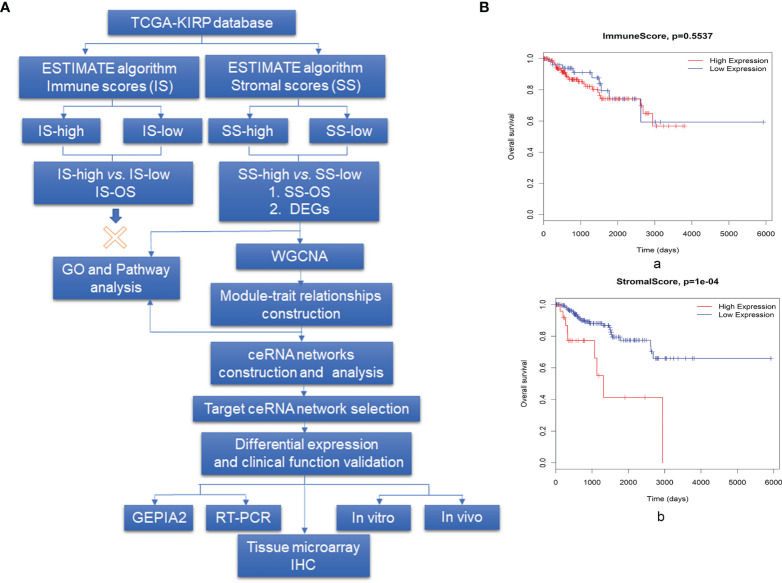
Overview of the data preparation, processing, analysis, and validation pipeline for this study and association between prognosis and both stromal scores (SCs) and immune scores (ISs). **(A)** Flowchart describing the bioinformatics analysis pipeline and experimental validation strategy in this study. (**B**, a) Survival analysis for patients in the IS-high and IS-low groups and (b) for patients in the SS-high and SS-low groups, using the R package Survival Analysis. High SSs are associated with a poor prognosis, whereas no survival difference was observed for those with high *vs.* low ISs.

We analyzed a patient cohort containing 233 KIRP patients for whom expression data were available in TCGA database. These data were analyzed using the ESTIMATE algorithm, as described in the flowchart in **Figure 1A**. Infiltrating cells in the tumor tissue were assessed by incorporating two gene signatures within the ESTIMATE algorithm. SSs were designed to capture the presence of stromal cells in tumor tissue, and ISs were calculated to determine the infiltration of immune cells in tumor tissue. Based on ISs, 233 samples were divided into an IS-high (178 samples) group and an IS-low (22 samples) group. We detected no difference in overall survival (OS) between patients in the IS-high and IS-low group (**Figure 1B, **a). We then divided patients into SS-high (27 samples) and SS-low (206 samples) groups, and found a significant difference in survival between patients in these groups, with SS-high patients showing decreased survival relative to SS-low patients (*P* = 1×10^-4^, **Figure 1B, **b). Based on these findings, we compared gene expression profiles in the SS-high and SS-low groups.

### Analysis of DEGs in SS-High vs. SS-Low Patients and WGCNA

**Figure 2:**
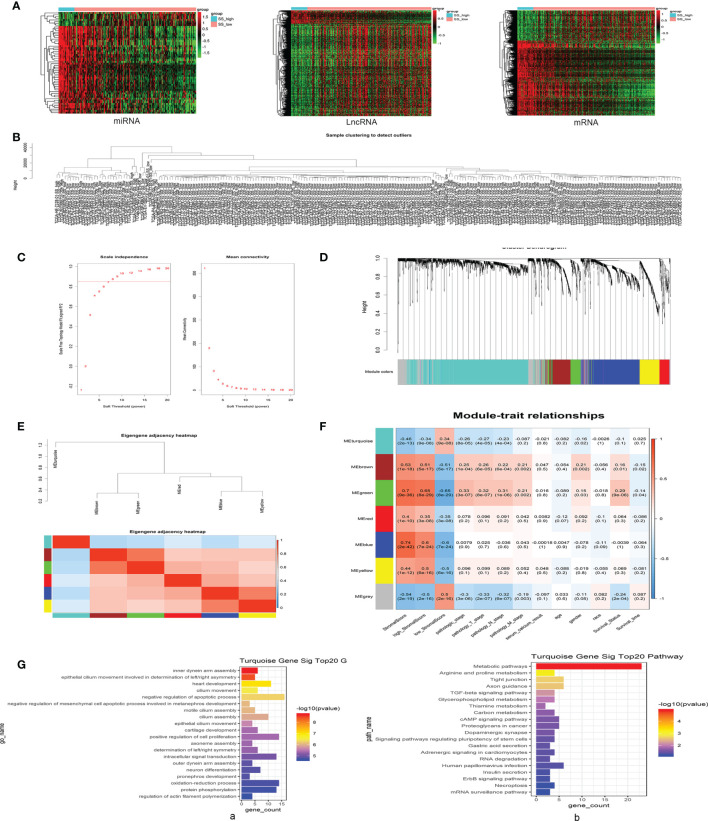
Differentially expressed genes (DEGs) in patients with high *vs.* low SSs were evaluated using weighted gene co-expression network analysis (WGCNA), and the turquoise module was selected based on positive pathological roles in KIRP. **(A)** Unsupervised hierarchical clustering analyses for DEGs, including differentially expressed messenger (m)RNAs, long noncoding (lnc)RNAs, and micro (mi)RNAs, in SS-high and SS-low KIRP tissue samples. The cluster analysis heat map shows the correlation between expression maps and group conditions. The rows represent differentially expressed miRNAs, lncRNAs, and mRNAs, and the columns represent the samples. **(B)** Sample clustering detection revealed no outlier samples. **(C)** Soft-threshold power (β) for co-expression of lncRNAs/mRNAs was determined by analyzing the network topology with a soft-threshold power ranging from 1 to 20. **(D)** Different modules were identified by the Dynamic Tree Cutting method, and each module was assigned a color as an identifier. Six modules were generated after merging based on the correlation of modules with WGCNA. **(E)** Heatmap plot of the adjacencies in the hub gene network; red represents positive correlation with high adjacency, and blue represents negative correlation with low adjacency. Squares of red color along the diagonal represent the meta-module. **(F)** Matrix of module–trait relationships and *P*-values for selected traits. Each column corresponds to a module eigengene, and each row corresponds to a histopathological trait. Each cell contains a corresponding correlation and *P*-value. The table is color-coded by correlation according to the color legend. **(G**, a**)** Gene ontology (GO) enrichment analyses of turquoise mRNAs; significant top 20 GO terms are shown. (**G**, b) Kyoto Encyclopedia of Genes and genomes (KEGG)-pathway enrichment analyses of turquoise mRNAs; significant top 20 signaling pathways are shown.

Identification of DEGs in KIRP samples with high *vs.* low SSs using the methods described above and in **Figure 1A** revealed 1668 differentially expressed mRNAs, 783 differentially expressed lncRNAs, and 58 differentially expressed miRNAs. All DEGs in each independent dataset were analyzed using unsupervised hierarchical clustering analysis. The cluster analysis heat map shows the correlation between expression maps and group conditions (**Figure 2A**). We further performed gene ontology (GO) and Kyoto Encyclopedia of Genes and Genomes (KEGG) pathway enrichment analyses of 1189 upregulated mRNAs in SS-high subjects and identified immune response and cytokine-cytokine receptor interactions among the top pathways. Analysis of 479 downregulated mRNAs yielded oxidation-reduction process and metabolic pathways as top hits (**Supplementary Figure 1**).

We next performed sample clustering detection of lncRNAs and mRNAs and found no outlier samples, indicating it was not necessary to remove any samples in our co-expression analyses (**Figure 2B**). The most important step in this process is selection of the value for soft-threshold power (β). To determine the relative equilibrium between scale independence and mean connectivity, we analyzed the network topology with soft-threshold power from 1 to 20, eventually confirming a β value of 8 for lncRNAs and mRNAs (**Figure 2C**). Next, we identified co-expression modules by hierarchical clustering and Dynamic Branch Cutting and assigned a unique color as an identifier in each module using WGCNA (**Figure 2D**).

To identify interactions among these co-expression modules, we analyzed the connectivity of eigengenes in a cluster analysis. Seven modules (turquoise, blue, grey, brown, yellow, green, red) were generated, with the grey module representing a gene set that was not assigned to any of the modules. We then generated a heatmap plot of the adjacencies in the hub gene network, wherein red represents positive correlation with high adjacency, and blue represents negative correlation with low adjacency (**Figure 2E**). The eigengene dendrogram and heatmap were used to identify groups of correlated eigengenes, and the dendrogram revealed a significant association between these modules and KIRP clinical traits. Notably, the turquoise module was found to be negatively correlated with pathological stage of the tumor and was chosen as the focus of research for this study (**Figure 2F**). This module contained a total of 1004 differentially expressed RNAs, including 364 mRNAs, 600 lncRNAs, and 40 other noncoding RNAs. GO enrichment analyses of the 364 mRNAs in the turquoise module identified negative regulation of apoptotic process as a top function, with the largest number of enriched genes (**Figure 2G,** a), and KEGG-pathway enrichment analyses found metabolic pathway as a key mechanism (**Figure 2G, **b).

### Selection of *CAMK2B* as a Key Gene Based on Correlation Strength and Survival Analyses With the Selected ceRNA Network

**Figure 3:**
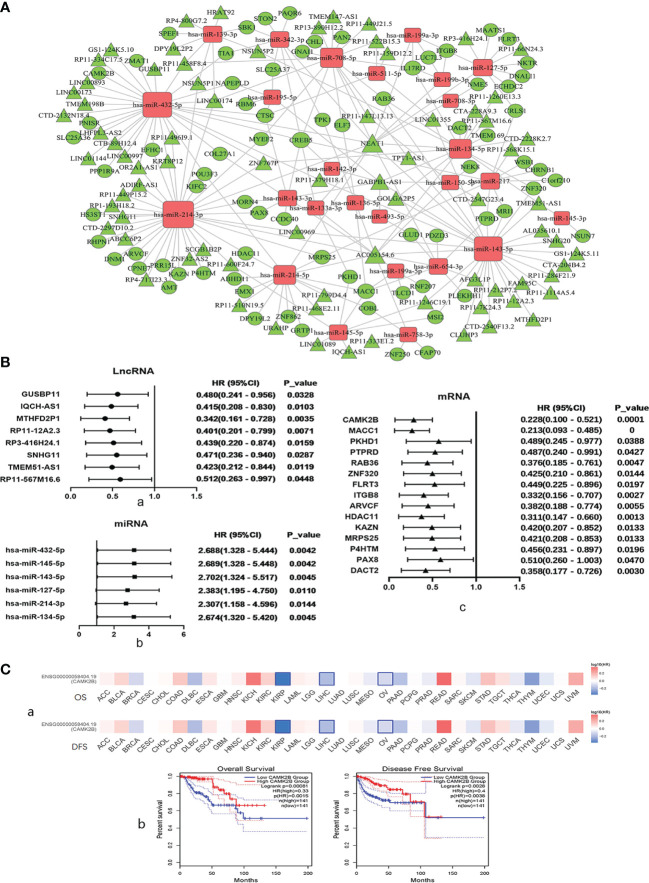
Regulation and co-expression of competing endogenous (ce)RNA networks in the turquoise module by WGCNA and selection of *CAMK2B* as a key gene based on results of correlation strength and survival analyses. **(A)** Co-expression of ceRNA networks in the turquoise module, including lncRNA GUSBP11/miR-432-5p/*CAMK2B*, was analyzed by WGCNA. Diamonds represent miRNAs, circles represent mRNAs, and cones represent lncRNAs. Upregulated genes are shown in red, and downregulated genes are shown in green. **(B)** Hazard ratios and corresponding 95% confidence intervals were estimated using the Cox proportional hazard regression model, and the prognostic effects of these (a) lncRNAs, (b) miRNAs, and (c) mRNAsare presented in a forest plot. (**C**, a) The prognostic impact of *CAMK2B* in 33 types of human tumors was analyzed using GEPIA2, revealing that high expression of CAMK2B is associated with poor prognosis in KIRP, liver hepatocellular carcinoma (LIHC), and ovarian cancer (OV). (b) Kaplan–Meier plotter was used to analyze survival, and high expression of CAMK2B was found to be associated with longer overall survival (OS) and recurrence-free survival (RFS).

Through our predefined strategy based on SSs, DEGs in the turquoise module were found to be mainly associated with favorable survival outcomes in KIRP patients. We then analyzed co-expression of ceRNA networks in the turquoise module by WGCNA and built multiple groups, including RP3-416H24.1/hsa-miR-127-5p/*ITGB8*, SNHG11/hsa-miR-214-3p/*ARVCF*, SNHG11/hsa-miR-214-3p/*HDAC11*, SNHG11/hsa-miR-214-3p/*KAZN*, SNHG11/hsa-miR-214-3p/*MRPS25*, SNHG11/hsa-miR-214-3p/*PRR15L*, and GUSBP11/miR-432-5p/*CAMK2B* (**Figure 3A**).

The prognostic effects of these DEGs were analyzed and presented in a forest plot, and eight lncRNAs, including GUSBP11, IQCH-AS1, MTHFD2P1, RP11-12A2.3, RP3-416H24.1, SNHG11, TMEM51-AS1, and RP11-567M16.6, were found to exhibit pro-survival functions with longer overall survival (OS) (**Figure 3B, **a). A total of seven miRNAs, including mir-432-5p, mir-145-5p, mir-143-5p, mir-127-5p, mir-214-3p, and mir-134-5p, were correlated with a poor prognosis and shorter OS (**Figure 3B, **b). In addition, 15 mRNAs were correlated with better prognosis, including *CAMK2B*, *MACC1*, *PKHD1*, *PTPRD*, *RAB36*, *ZNF320*, *FLRT3*, *ITGB8*, *ARVCF*, *HDAC11*, *KAZN*, *MRPS25*, *P4HTM*, *PAX8*, and *DACT2* (**Figure 3B, **c). We then plotted Kaplan–Meier curves using the Cox proportional hazard regression model for SS-related lncRNAs, miRNAs, and mRNAs in KIRP and confirmed that all these genes are associated with survival (**Supplementary Figure 2**). Based on results of correlation strength and survival analyses, *CAMK2B* was selected as one of the most promising target genes.

We then assessed the prognostic impact of *CAMK2B* in 33 types of human tumors using GEPIA2 (12), and found that high expression of *CAMK2B* is associated with improved prognosis in KIRP, liver hepatocellular carcinoma (LIHC), and ovarian cancer (OV, **Figure 3C, **a). To further confirm this finding, we used Kaplan–Meier plotter and found that that high expression of *CAMK2B* is associated with longer OS and recurrence-free survival (RFS) in KIRP (**Figure 3C, **b).

### Expression and Clinical Role of CAMK2B in KIRP Samples

**Figure 4:**
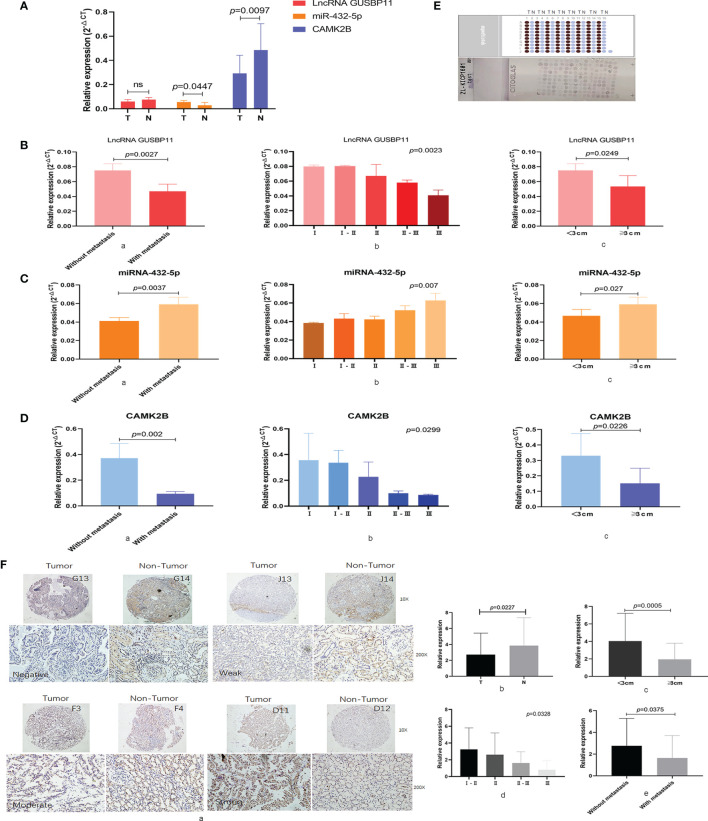
Expression and clinical role of CAMK2B and its associated ceRNA network in KIRP samples. **(A)** Expression of the selected ceRNA network, including *CAMK2B*, mir-432-5p, and lncRNA GUSBP11, was measured using quantitative reverse transcription (qRT)-PCR in KIRP tissue and normal tissue adjacent to the carcinoma. Ns, no significance. (**B**, a) In cancer patients, high expression of lncRNA GUSBP11 is associated with no metastasis, (b) early-stage disease, and (c) small tumor size. (**C**, a) High expression of mir-432-5p was associated with tumor metastasis, (b) late-stage disease, and (c) large tumor size. (**D**, a) High expression of *CAMK2B* is associated with no metastasis, (b) early-stage disease, and (c) small tumor size. **(E)** Protein levels of CAMK2B were measured by immunohistochemistry (IHC) using tissue microarrays. (**F **a, b) Protein levels of CAMK2B are lower in tumor tissue than in paired tumor-adjacent tissue. (c) Expression of CAMK2B is higher in tumors <3 cm in size than in tumors >3 cm in size. (d) High expression of CAMK2B is positively correlated lower tumor grade. (e) High expression of CAMK2B is negatively correlated with tumor metastasis.

We next measured expression of the selected ceRNA network in KIRP tissue by qRT-PCR. We detected lower levels of lncRNA GUSBP11 (*P*=0.0618) and *CAMK2B* (*p*=0.0447) in tumor tissue samples than in the normal tissue adjacent to the carcinoma, whereas levels of miR-432-5p were found to be higher in tumor tissue (*P*=0.0097; **Figure 4A**). Upon further exploration, we found that high expression of lncRNA GUSBP11 is associated with no metastasis (*P*=0.0023; **Figure 4B, **a), early-stage disease (*P*=0.0023; **Figure 4B, **b), and small tumor size (*P*=0.0249; **Figure 4B, **c). Similarly, high expression of *CAMK2B* mRNA is associated with no metastasis (*P* = 0.002; **Figure 4D, **a), early-stage disease (*P*=0.0299; **Figure 4D, **b), and small tumor size (*P*=0.0226; **Figure 4D, **c). In contrast, high expression of mir-432-5p is associated with tumor metastasis (*P*=0.0037; **Figure 4C, **a), late-stage disease (*P*=0.007; **Figure 4C, **b), and large tumor size (*P*=0.027; **Figure 4C, **c).

We further confirmed these findings by analyzing KIRP tumor samples and adjacent non-tumor tissues by IHC using tissue microarray (**Figure 4E, F**). Indeed, we found that protein levels of CAMK2B are lower in tumor tissue than in paired tumor-adjacent tissue (*P*=0.0227, **Figure 4F, **a, b). Further, expression of CAMK2B in tumors <3 cm in size is significantly higher than in tumors >3 cm in size (*P*=0.0005, **Figure 4F,** c, Confirm whether the insertion of the Ethics Statement section is fine. Note that we have used the statement provided at Submission. If this is not the latest version, please let us know.). High expression of CAMK2B was also found to be associated with lower tumor grade (*P*=0.0328, **Figure 4F, **d) and low metastasis (*P*=0.0375, **Figure 4F, **e). Thus, these data confirm the expected pattern of CAMK2B expression and suggest it acts as a protective factor in KIRP.

### Upregulation of *CAMK2B* Is Associated With Decreased Proliferation and Inhibition of the Tumor Stromal Microenvironment in KIRP

**Figure 5:**
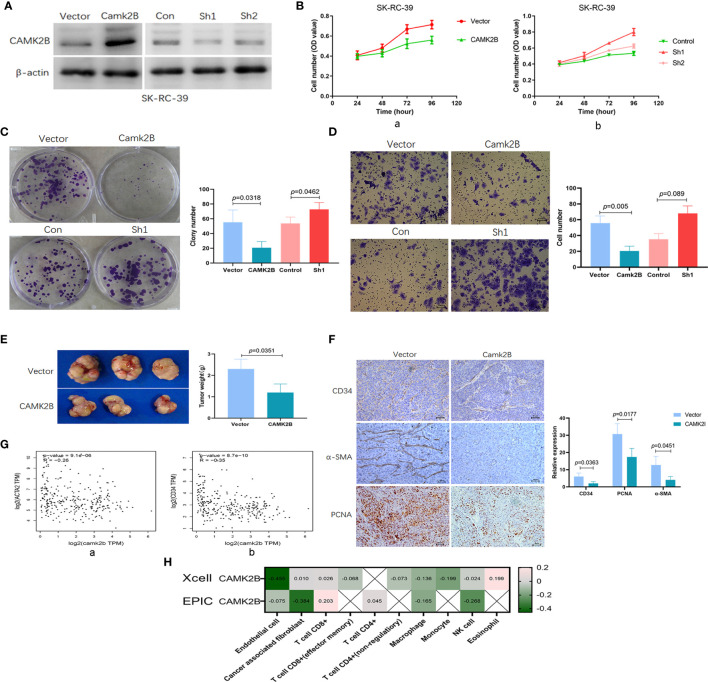
Upregulation of CAMK2B is associated with decreases in proliferation and fibroblast infiltration, as well as inhibition of angiogenesis. **(A)** CAMK2B was overexpressed or subjected to by short-hairpin (sh) RNA-mediated silencing in SK-RC-39 cells using lentiviral transfection. **(B)** CAMK2B inhibits cell proliferation (a), and this is reversed by CAMK2B silencing, as determined using the CCK8 assay (b). **(C)** Upregulation of CAMK2B inhibits cell proliferation ability in a sphere formation assay (a), and this is reversed by CAMK2B silencing (b). **(D)** CAMK2B inhibits cell migration ability (a), whereas migration is enhanced after CAMK2B silencing (b), as measured using the Boyden Chamber migration assay. **(E)** Diminished subcutaneous tumor growth is observed in mice injected with SK-RC-39-CAMK2B cells relative to those injected with SK-RC-39 cells. **(F)** Immunohistochemical staining confirms significantly decreased expression of CD34, α-SMA, and PCNA in subcutaneous tumor tissues derived from SK-RC-39-CAMK2B cells *vs.* tissue from control cells. **(G)** Negative correlation is observed between expression of *CAMK2B* and both α-SMA and CD34 in human KIRP tissue from TCGA database using GEPIA2. **(H)** Significant negative correlations are observed between expression of CAMK2B and both endothelial cells (R=-0.455) and cancer-associated fibroblasts (R=-0.384) using Xcell and EPIC, whereas the correlation with immune cells is weak.

To evaluate the function of CAMK2B, we stably overexpressed and silenced *CAMK2B* expression in the KIRP SK-RC-39 cell line (**Figure 5A**). We found that upregulation of *CAMK2B* significantly inhibits cell proliferation (**Figure 5B, **a), whereas the reverse occurs in response to *CAMK2B* silencing (**Figure 5B, **b). Moreover, we confirmed the inhibition of proliferation in response to CAMK2B overexpression using sphere formation assays, and showed that *CAMK2B* silencing has the opposite effect (**Figure 5C**). Migration assays further showed that a lower number of *CAMK2B* overexpressing cells cross the basement membrane relative to SK-RC-39 control cells, whereas migration is enhanced after *CAMK2B* silencing (**Figure 5D**).

In nude mouse models, diminished subcutaneous tumor growth was observed in mice injected with SK-RC-39 cells stably overexpressing *CAMK2B* (*P*=0.0351, **Figure 5E**) as compared to mice injected with SK-RC-39 control cells. Immunohistochemical staining further revealed that SK-RC-39-CAMK2B subcutaneous tumors from these animals show significantly decreased expression of CD34 (*P*=0.0363), α-SMA (*P*=0.0451), and PCNA (*P*=0.0177, **Figure 5F**) relative to SK-RC-39 tumors. We also confirmed this negative correlation between expression of CAMK2B and both α-SMA (R=-0.26, **Figure 5G, **a) and CD34 (R=-0.35, **Figure 5G, **b) in human KIRP tissue using the GEPIA2 database (12), indicating that elevated CAMK2B levels are associated with decreased infiltrating fibroblasts and vascular endothelial cells. Additionally, the negative correlation between expression of CAMK2B and endothelial cells (R=-0.455) and cancer-associated fibroblast (R=-0.384) was confirmed using Xcell and EPIC. In contrast, only a weak correlation is observed between CAMK2B and immune cells (**Figure 5H**). Thus, the above findings suggest that upregulation of CAMK2B decreases proliferation and inhibits tumor stromal cell infiltration in KIRP.

### CHL1, VEGF, and TGFβ Act as Downstream Effectors of CAMK2B in KIRP

**Figure 6:**
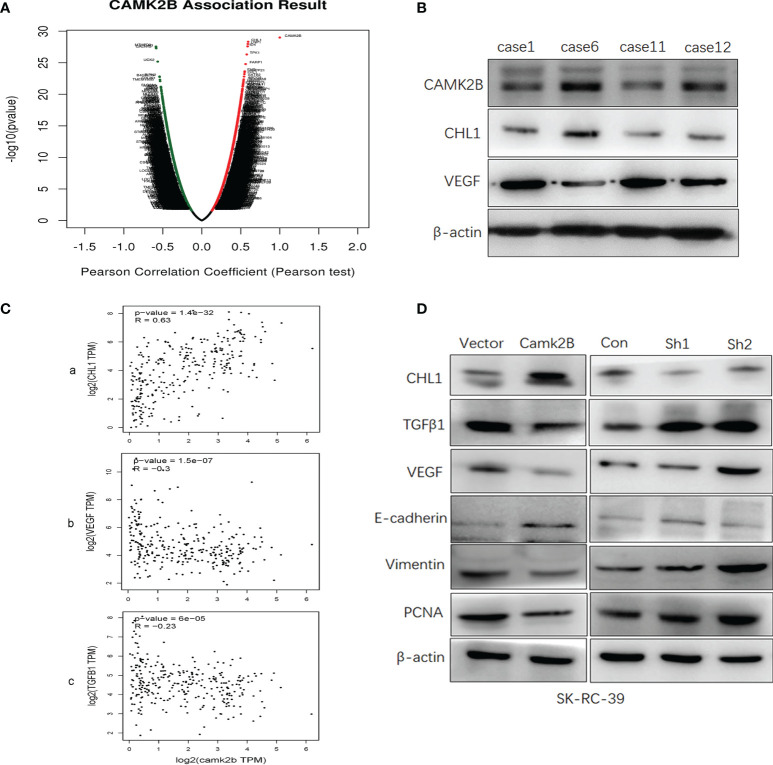
CHL1, VEGF, and TGFβ act as downstream effectors of CAMK2B. **(A)** Genes co-expressed genes with CAMK2B in KIRP tissue were screened in TCGA database using LinkedOmics and Metascape and visualized in a volcano plot. **(B)** The protein levels of CAMK2B, CHL1, and VEGF in tissue from KIRP patients were evaluated by immunoblotting. **(C)** The correlation between the expression levels of *CAMK2B* and *CHL1* (a), *CAMK2B* and *CHL1* (b), and *CAMK2B* and *TGFβ1* (c) were evaluated in TCGA database using GEPIA2. **(D)** Expressed of several predicted effector molecules, including TGFβ1, VEGF, vimentin, PCNA, CHL1, and E-cadherin were measured in KIRP cells overexpressing or silenced for *CAMK2B*.

In order to identify potential target genes downstream of CAMK2B, we screened for CAMK2B co-expressed genes in KIRP from TCGA database using LinkedOmics (13) and Metascape (14). We then constructed a volcano plot to visualize genes that are positively and negatively correlated with CAMK2B and identified CHL1 as the most positively correlated gene and VEGFA as the most negatively correlated gene (**Figure 6A**). Using Metascape, we found that the top 100 genes correlated with CAMK2B are significantly enriched in cellular component organization and biogenesis (**Supplementary Figure 3**). To confirm the relationship between CAMK2B, VEGFA, and CHL1 in KIRP, we measured the levels of these proteins in frozen tissue samples from KIRP patients by immunoblotting, revealing a positive correlation between CAMK2B and CHL1 and a negative correlation between CAMK2B and VEGF (**Figure 6B**). Further evaluating their expression in KIRP tissue using GEPIA2 confirmed a significant positive correlation between expression levels of *CAMK2B* and *CHL1* (R=0.7433), as well as a negative correlation between expression of *CAMK2B* and both *VEGF* (R=-0.3) and *TGFβ1* (R=-0.23, **Figure 6C**). Finally, we examined expression of several effector molecules in SK-RC-39 cells stably overexpressing *CAMK2B* or subjected to shRNA-mediated *CAMK2B* silencing. Notably, we found that expression of CHL1 and E-cadherin are significantly increased, whereas TGFβ1, VEGF, vimentin, and PCNA are significantly downregulated, in SK-RC-39 cells overexpressing CAMK2B. Conversely, when CAMK2B expression in SK-RC-39 cells is silenced using specific shRNA, the opposite expression pattern is observed (**Figure 6D**). Thus, we identify CHL1, VEGF, and TGFβ1 as downstream effectors of CAMK2B, and provide further evidence that enhanced CAMK2B expression inhibits proliferation and stromal cell infiltration.

## Discussion

Renal papillary cell carcinoma is insensitive to conventional radio- and chemotherapies, and consequently, surgical excision is the only useful clinical intervention that demonstrably prolongs patient survival in early-stage disease. For unresectable KIRP at an advanced stage, targeted therapy [ e.g., tyrosine kinase inhibitors [TKIs)] is recommended (15). However, resistance to targeted therapy, including adaptive resistance, intrinsic resistance, and acquired resistance, remain major obstacles to treatment success (16, 17). The emergence of immune checkpoint inhibitors has further provided hope to advanced-stage KIRP patients, and studies aimed exploring strategies for immunotherapy, either alone or in combination with TKI inhibitors, in KIRP patients are urgently needed. In parallel, much remains unknown about the tumor microenvironment and molecular characteristics in KIRP, and this is therefore an important area of investigation.

During cancer progression, a series of genetic and phenotypic changes occur that, in addition to tumor-cell derived cytokines, chemokines, and metabolites, have been reported to significantly impact the TME (18). The TME is mainly composed of various immune cells, vascular endothelial cells, and fibroblasts, which generate permissive niches for tumor progression (19). Heterogeneous features of the TME determine both the diverse fates and drug responses of tumors, and the TME also has strong predictive value for tumor prognosis (20). In particular, the importance of immune cell populations, reflecting the capacity of the immune system to sense tumor cells, has been recognized for prognosis (21). The field of cancer immunotherapy is evolving rapidly. Notably, response to immunotherapy has been shown to be dynamically regulated by the TME *via* cell-cell interactions and paracrine signals emanating from the tumor. Ipilimumab, a monoclonal antibody against CTLA-4, demonstrates response rates between 5–12.5%, but no complete responses or durable regressions were seen in renal cell carcinoma (22). Nivolumab, a monoclonal antibody that blocks the PD-1 pathway demonstrated complete responses in only 1% of renal cell carcinoma patients (23). Zhou *et al.* found increased number of tumor-infiltrating lymphocytes in high-risk KIRP patients based on expression 14 immune-related genes, but detailed materials revealed that total levels of immune cells in tumor tissues were not high (24). In this study, we surprisingly found no difference in survival for patients with high *vs.* low IS, suggesting a rationale for why immunotherapy is not effective in KIRP patients.

Tumors are supported by a complex microenvironment that is characterized by the presence of many stromal cell populations. For example, fibroblasts and endothelial cells create a desmoplastic tumor niche that plays an essential role in malignant tumor progression (25, 26). Therefore, we further explored the factors that influence KIRP progression from this perspective. To this end, we divided patients into SS-high and SS-low groups and found a significant difference in survival between patients with high *vs.* low SS, suggesting that non-immune stromal cells may play important roles in tumor progression. We further identified 1668 mRNAs, 783 lncRNAs, and 58 miRNAs that are differentially expressed in SS-high compared to SS-low tumors and analyzed these three independent datasets by WGCNA. Using these data, we then constructed co-expression networks of ceRNAs. Previous studies have demonstrated that ceRNAs affect the proliferation, growth, differentiation, apoptosis and other biological behaviors of cancer cells (27). In this study, we focused on co-expression ceRNA networks in the turquoise module, as this was found to be negatively correlated with pathological stage of the tumor.

We evaluated the expression and clinical roles for the members of the selected ceRNA network lncRNA GUSBP11/miR-432-5p/*CAMK2B* and identified a close correlation with KIRP patient survival, suggesting that the effector gene *CAMK2B* may be leveraged for prognostic and therapeutic purposes. CAMK2B belongs to the serine/threonine protein kinase family and to the Ca(2+)/calmodulin-dependent protein kinase subfamily. This protein was shown to promote neuronal survival (28), although a role in cancer has not been demonstrated. Here, to evaluate the precise function of CAMK2B, we stably overexpressed and silenced *CAMK2B* expression in a KIRP cell line and found that upregulation of CAMK2B inhibits both proliferation and tumor stromal cell infiltration *in vivo* and *in vitro*. We then screened for genes co-expressed with CAMK2B in KIRP tissues using Metascape and identified *CHL1* as a putative CAMK2B target gene with the highest correlation. CHL1 is a member of the L1 gene family of neural cell adhesion molecules, which may also play negative role in the growth of certain cancers (29, 30). In this study, we found that expression of CAMK2B and CHL1 is positively correlated with resisting cell death, and negatively correlated with induction of angiogenesis and fibrogenesis. We therefore propose that CHL1 acts downstream of CAMK2B, and this mechanism may represent one of the targetable nodes in KIRP patients. VEGF and TGFβ1 have shown positive correlation with cancer (31), and the expression of both are negative correlated with CAMK2B, suggesting they also act as downstream effectors of CAMK2B in KIRP.

In summary, here we show that the SS-related network lncRNA GUSBP11/miR-432-5p/*CAMK2B* exhibits prognostic potential in KIRP, and CAMK2B may represent an effective therapeutic target. The cellular mechanism of action for CAMK2B mainly relates to inhibiting proliferation and remodeling angiogenesis and fibrogenesis. Thus, we further speculate that anti-fibrosis or anti-angiogenic therapy could be a better therapeutic approach than immunotherapy for KIRP, with CAMK2B as one possible target. However, several fundamental questions remain to be answered concerning the ability of CAMK2B to remodel stromal TMA. Therefore, cirrhosis mouse models with a blood vessel-rich background should be established to further elucidate the relationship among CAMK2B, fibroblasts, and vascular endothelial cells. In future studies, we will also determine if anti-angiogenesis therapy shows efficacy for clinical treatment of KIRP.

## Data Availability Statement

The original contributions presented in the study are included in the article/**Supplementary Material**. Further inquiries can be directed to the corresponding author.

## Ethics Statement

The studies involving human participants were reviewed and approved by the ethics committee on Human Research of Tangdu Hospital. The patients/participants provided their written informed consent to participate in this study. The animal study was reviewed and approved by the Medical Experimental Animal Care Commission of Northwest Polytechnical University. Written informed consent was obtained from the individual(s) for the publication of any potentially identifiable images or data included in this article.

## Author Contributions

All authors searched the literature, designed the study, interpreted the findings, and revised the manuscript. XF and QJ conceived the study. BX, YS and GB performed the bioinformatics analyses. YZ and XL performed the majority of experiments. QJ and XL participated in data analysis and visualization. QJ drafted and prepared the manuscript. All authors contributed to the article and approved the submitted version.

## Funding

This research project was partly supported by the Natural Science Basic Research Program of Shaanxi (2021JM-080 and 2020JQ-230), Fundamental Research Funds for the Central Universities (31020200QD032). The funders did not play any role in manuscript design, data collection, data analysis, data interpretation, or writing of the manuscript.

## Conflict of Interest

The authors declare that the research was conducted in the absence of any commercial or financial relationships that could be construed as a potential conflict of interest.

## Publisher’s Note

All claims expressed in this article are solely those of the authors and do not necessarily represent those of their affiliated organizations, or those of the publisher, the editors and the reviewers. Any product that may be evaluated in this article, or claim that may be made by its manufacturer, is not guaranteed or endorsed by the publisher.
