# Supplementary figures and images for: Anti-Tumor Role of CAMK2B in Remodeling the Stromal Microenvironment and Inhibiting Proliferation in Papillary Renal Cell Carcinoma
Source: Front Oncol. 2022 Jan 21;12:740051. doi: 10.3389/fonc.2022.740051 (PMC8815460; doi:10.3389/fonc.2022.740051)

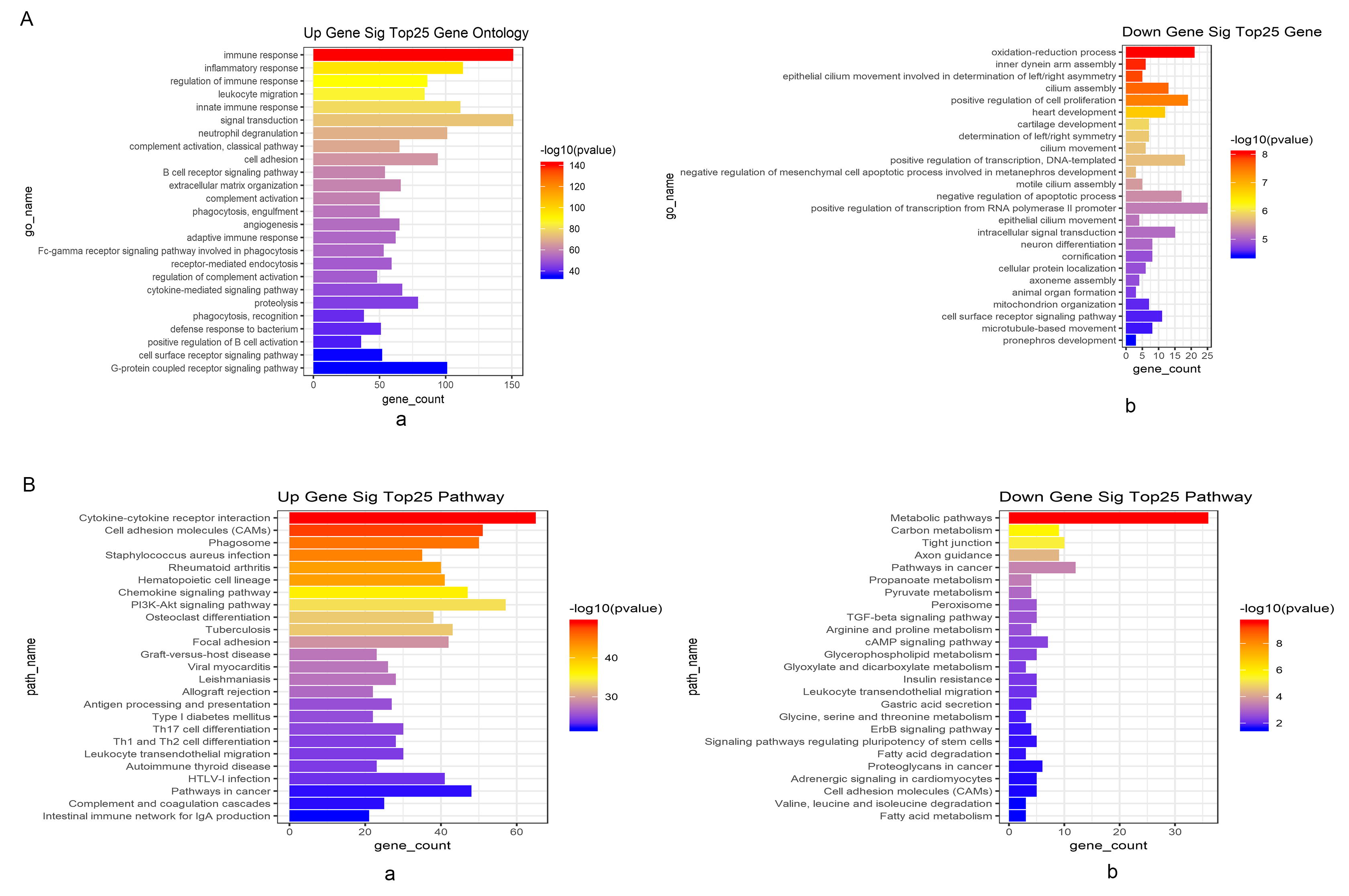

Supplement: Supplementary file 1 [file Image_1.tif]

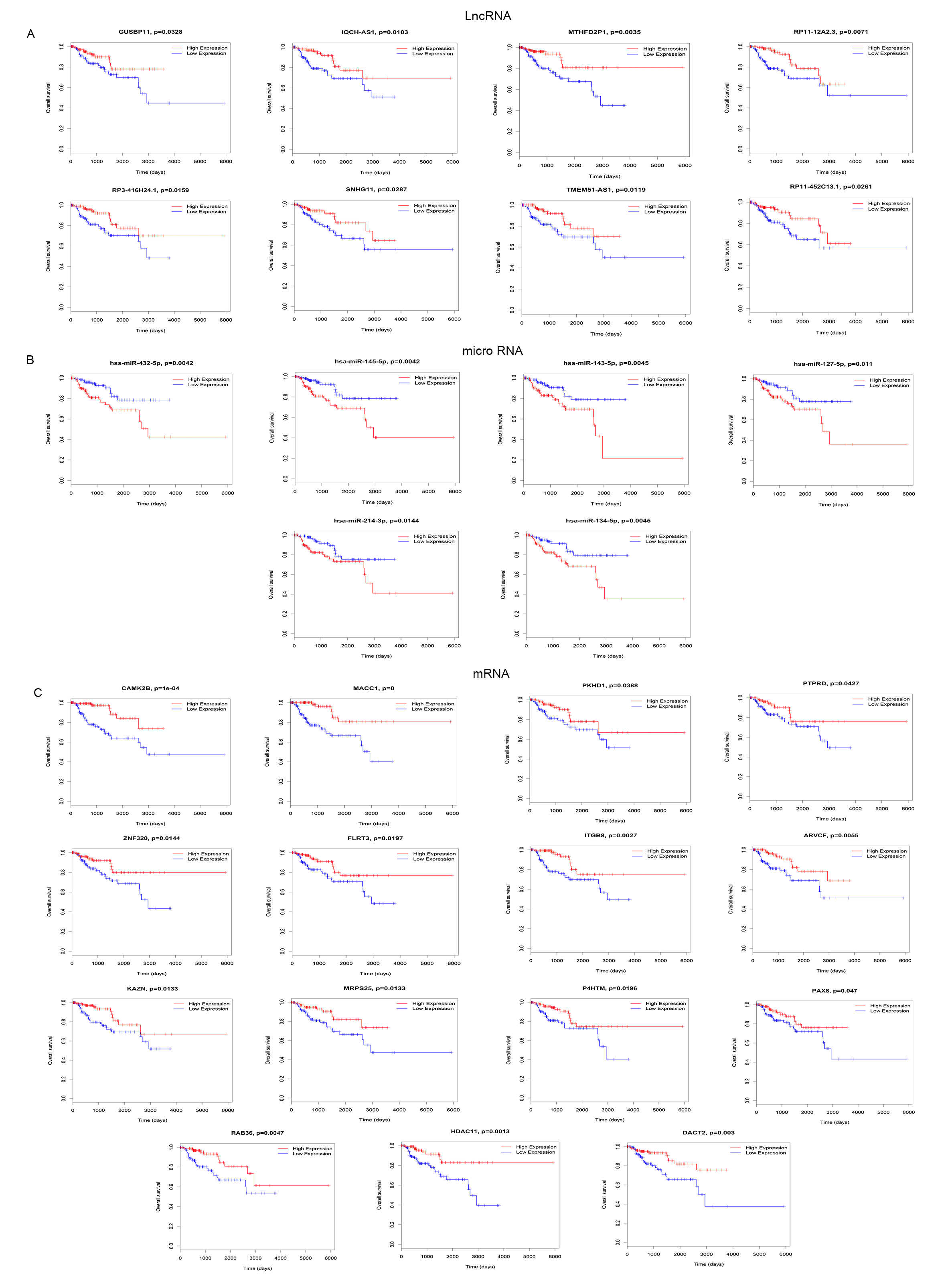

Supplement: Supplementary file 2 [file Image_2.tif]

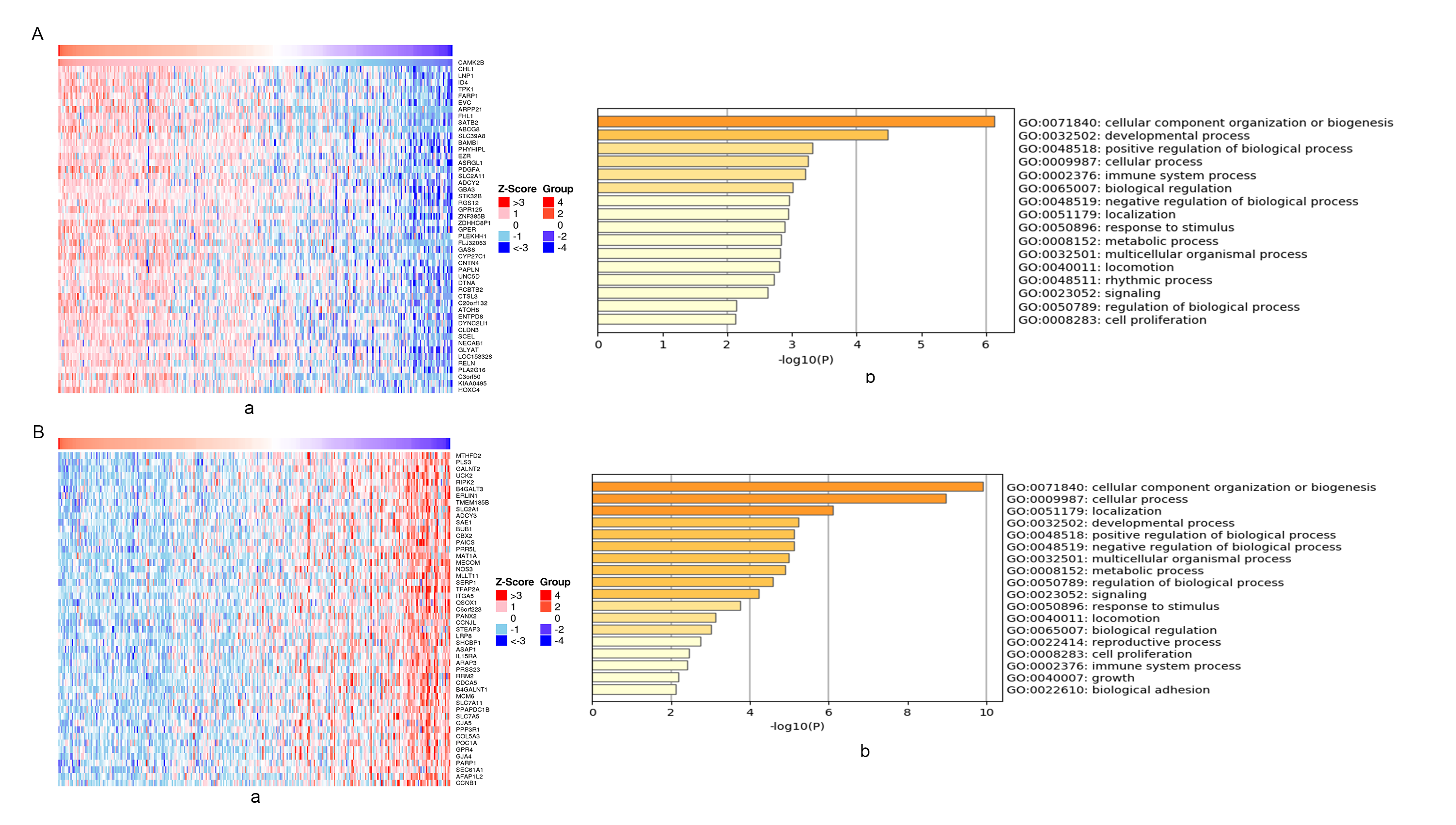

Supplement: Supplementary file 3 [file Image_3.tif]
