# Supplementary material for: Anti-Tumor Role of CAMK2B in Remodeling the Stromal Microenvironment and Inhibiting Proliferation in Papillary Renal Cell Carcinoma
Source: Front Oncol. 2022 Jan 21;12:740051. doi: 10.3389/fonc.2022.740051 (PMC8815460; doi:10.3389/fonc.2022.740051)
Supplement: Supplementary file 4 [file Table_1.docx]

**SUPPLEMENTARY MATERIALS AND METHODS**

**DEG extraction, GO and Pathway analysis**

We performed random variance model (RVM) *t*-test to filter the DEGs for the control and experimental groups. After performing significance and false discovery rate (FDR) analysis, we selected the DEGs according to the *p*-value threshold [1]. We performed hierarchical clustering using EPCLUST. GO analysis was applied to analyze the main function of the DEGs according to the gene ontology [2]. Specifically, two-side Fisher’s exact test and$\chi^{2}$ test were used to classify the GO category, and the FDR [3] was calculated to correct the *p*-value. Pathway analysis was used to find out the significant pathway of the differential genes according to KEGG. We continued to use the Fisher’s exact test and $\chi^{2}$ test to select the significant pathway, and the threshold of significance was defined by *p*-value and FDR. The enrichment (Re) was calculated using the equation ($\mathrm{Re}=(n_{f}/n)/(N_{f}/N)$) [4-6].

**Relationship between clinical information and modules**

We identified the correlation between modules and clinical information (i.e., stage, gender, race, survival time, etc.) in KIRP patients by Pearson's correlation coefficient (PCC). Above all, module eigengenes (MEs) referred to the first principal component of all gene expression levels in the module, and therefore, it was reasonable to consider that MEs represented all genes within a specific module. According to PCC, we further identified the association between MEs and external clinical information including sample status. *p*-value < 0.05 was considered to be a significant correlation.

**ceRNA network construction and analysis**

The interactions among lncRNAs, miRNAs, and mRNAs contribute to the post-transcriptional regulation of mRNAs. The associated ceRNA network in KIRP was constructed following three stages. (1) The miRNA-lncRNA and miRNA-mRNA relationships were collected in the starBase v2.0 database (<http://starbase.sysu>. edu.cn/); (2) mi-Randa tools (http://www.microrna.org/microrna/home.do), the miRNAs target prediction tool, were employed to detect the lncRNA-miRNA interactions. Finally, we analyzed the mRNA-miRNA interactions by miRTarBase (http://mirtarbase. mbc.nctu.edu.tw/) and TargetScan (http://www.targetscan.org/). (3) The ceRNA network was built and illustrated using Cytoscape v3.0.

**Antibodies and Cell lines**

Monoclonal antibodies were used for immunoblot and/or Immunohistochemistry analysis, including CAMK2B, α-SMA, E-cadherin, and Vimentin, PCNA, CHL1 (Abcam, Cambridge, MA, USA), HMGB1, Snail, and β-Actin (Proteintech, Chicago, USA).

The KIRP cell line SK-RC-39 was obtained from the the Air Force Military Medical University (Xi’ an, China), and cultured in Dulbecco’s Modified Eagle’s Media (DMEM; GIBCO, Grand Island, NY, USA) containing 10% foetal bovine serum (FBS; GIBCO) at 37°C in a humidified incubator with 5% CO_2_.

**Animal models**

A total of 6 male BALB/c nu/nu mice (aged 4–6 weeks and weighing approximately 20 g) and raised in a controlled environment with 25°C under standard pathogen-free conditions and a natural light/dark cycle (morning 8:00; afternoon 8:00), and were provided with water and standard diet. SK-RC-39-Vector, SK-RC-39-CAMK2B cells were implanted subcutaneously into the upper left flank region of mice to establish subcutaneous xenografts. Tumour weights were evaluated 5 weeks after the treatments. The intraperitoneal injection of pentobarbital (5 mg/kg) combined with cervical spondylolisthesis was used for euthanasia of the mice after the study. The study protocol was approved by the Medical Experimental Animal Care Commission of Northwest Polytechnical University, and all methods were performed in accordance with the relevant guidelines.

**Migration assays**

Migration of SK-RC-39 cells were evaluated in Boyden chambers containing membranes with 8.0-μm pores in 24-well plates (Corning, Tewksbury, MA, USA). Cells were seeded into the upper chamber of each well in serum-free DMEM (6×10^4^ cells/well). DMEM containing 2% FBS was added to the lower chamber of each well. After 24 h, cells on the underside of the membrane were stained with Giemsa (Sigma Chemical Company, St. Louis, MO, USA), counted, and photographed at 200× magnification.

**Proliferation assessment**

To investigate the proliferative effect of CAMK2B on KIRP cells, SK-RC-39 cells with overexpression or silenced CAMK2B (sh1 and sh2) (1×10^3^ cells/well) were plated in 6-well plates and cultured with DMEM containing 10% FBS. Culture medium was replaced every 3 d, and the colonies were fixed with ice-cold 4% paraformaldehyde after 14 d. Cells were stained with Giemsa (Sigma, St. Louis, MO, USA) and photographed at ×5 magnification.

SK-RC-39 cells with overexpression or silenced CAMK2B (sh1 and sh2) were plated in 96-well plates (3×10^3^ cells/well) and cultured for 24, 48, 72, and 96 h. Cell proliferation assays were carried out with the Cell Counting Kit 8 (CCK8; Dojindo). Results were expressed as the absorbance of each well at 450nm (OD450).

**Reference**

1. Clarke R, Ressom HW, Wang A, Xuan J, Liu MC, Gehan EA, Wang Y: **The properties of high-dimensional data spaces: implications for exploring gene and protein expression data**. *Nat Rev Cancer* 2008, **8**(1):37-49.

2. Gene Ontology C: **The Gene Ontology (GO) project in 2006**. *Nucleic Acids Res* 2006, **34**(Database issue):D322-326.

3. Dupuy D, Bertin N, Hidalgo CA, Venkatesan K, Tu D, Lee D, Rosenberg J, Svrzikapa N, Blanc A, Carnec A *et al*: **Genome-scale analysis of in vivo spatiotemporal promoter activity in Caenorhabditis elegans**. *Nature biotechnology* 2007, **25**(6):663-668.

4. Kanehisa M, Goto S, Kawashima S, Okuno Y, Hattori M: **The KEGG resource for deciphering the genome**. *Nucleic acids research* 2004, **32**(Database issue):D277-280.

5. Yi M, Horton JD, Cohen JC, Hobbs HH, Stephens RM: **WholePathwayScope: a comprehensive pathway-based analysis tool for high-throughput data**. *BMC bioinformatics* 2006, **7**:30.

6. Draghici S, Khatri P, Tarca AL, Amin K, Done A, Voichita C, Georgescu C, Romero R: **A systems biology approach for pathway level analysis**. *Genome research* 2007, **17**(10):1537-1545.
